# Characterisation of iatrogenic human ureteral laser injury - results from ex vivo testing with a Ho: YAG laser at commonly used lithotripsy settings

**DOI:** 10.1007/s00345-025-05713-2

**Published:** 2025-06-04

**Authors:** S. O’Meara, K. Sheehan, J. Fay, T. O’Grady, S. M. Croghan, E. M. Cunnane, D. Hogan, G. G. Calpin, B. Somani, M. T. Walsh, F. J. O’Brien, N. F. Davis

**Affiliations:** 1 Strategic Academic Recruitment (StAR) Programme, Royal College of Surgeons in Ireland, Dublin, Ireland; 2 Dept. of Urology, Blackrock Health, Blackrock, Dublin, Ireland; 3 Dept. of Pathology, Royal College of Surgeons in Ireland, Dublin, Ireland; 4 Dept. of Pathology, Blackrock Health, Dublin, Ireland; 5 RCSI biobank, Dept. of Pathology, Royal College of Surgeons in Ireland, Dublin, Ireland; 6 Dept. of Urology, Beaumont Hospital, Dublin, Ireland; 7 Biomaterials Cluster, Bernal Institute, University of Limerick, Limerick, Ireland; 8 School of Engineering, University of Limerick, Limerick, Ireland; 9 Tissue Engineering Research Group, Royal College of Surgeons in Ireland, Dublin, Ireland; 10 Department of Urology, University Hospital Southampton NHS Foundation Trust, Southampton, SO16 6YD UK

**Keywords:** Urolithiasis, Laser, Endourology, Iatrogenic injury, Stricture

## Abstract

**Introduction:**

Modern endourology relies on Ho: YAG lasers for definitive management of urolithiasis. Current safety data is drawn predominantly from Ho: YAG laser testing in animal tissue. We aim to evaluate and characterise the grades of iatrogenic human ureteral injury due to contact with a Ho: YAG laser at commonly used lithotripsy settings.

**Methodology:**

Human ureters were collected from consenting adults at the time of radical nephrectomy. A custom rig was developed to secure a 273micron (µm) laser fibre in contact with the urothelium for periods of 5, 10 and 20 s. Testing settings used included 8 Hz (Hz) and 800millijoules (mJ), 10 Hz/1000 mJ, and 5 Hz/1500 mJ. Grades of ureteric injury were then characterised with histopathology.

**Results:**

A total of 31 specimens were tested and the mean specimen thickness was 2.2 mm (range 0.9–3.1 mm). The depth of laser-related injury ranged from urothelium alone to full thickness perforation at all settings depending on the duration of laser contact with tissue and specimen thickness. No full thickness perforation was seen in specimens ≥ 3 mm, even at 20 s of contact duration at 10 Hz/1000 mJ. However, in specimens < 1 mm the level of injury depth ranged from urothelium to full thickness perforation at 5 s of testing.

**Discussion:**

Ho: YAG laser causes variable levels of injury, with both superficial and extensive injury noted. The thickness of the ureter may determine the extent of trauma following laser contact. Concerningly, full thickness ureter perforation may occur with commonly used laser settings at short laser contact durations.

## Introduction

The holmium: yttrium-aluminium-garnet (Ho: YAG) surgical laser has a defined role in lithotripsy and tissue ablation in endourology. Its depth of tissue penetration is approximately 0.4 millimetres (mm) which contributes to the laser’s favourable safety profile, with clinical studies reporting low rates of tissue trauma [1–7]. Despite its common use, there is ongoing debate regarding the optimal Ho: YAG settings for efficient stone or tissue destruction while ensuring patient safety [8–12]. Recent ex-vivo animal studies have suggested that the actual depth of tissue trauma is deeper than the penetration depth previously described [6, 13]. 

Studies evaluating the detrimental impact of Ho: YAG laser contact with human ureteric tissue are lacking, with available safety data relying on experiments in animal tissue [6, 9, 13–16]. One previous study in ex-vivo human ureter tissue evaluated the time to visual perforation when the laser fibre was in tangential contact with the specimen [17]. The objective of the present study is to evaluate and characterise the histological grades of iatrogenic human ureteral injury due to contact from the Ho: YAG laser at commonly used lithotripsy settings.

## Methodology

### Overview of experimental design

Ex-vivo testing of Ho: YAG laser was performed in direct contact with human ureteric tissue to evaluate the impact of commonly used laser settings, duration of contact and specimen characteristics on the level of laser injury. Following development and testing of an experimental protocol, a custom rig was developed to simulate clinical settings. The ureter was tested within one hour of collection, and testing was performed in a water bath at physiological temperature, with the laser in direct contact with the ureter. Histological review of specimens was used to determine the extent of laser injury and to evaluate for perforation of the ureter. Perforation was defined as trauma extending through all ureteric anatomical layers including the adventitia.

### Specimen preparation

**Fig. 1:**
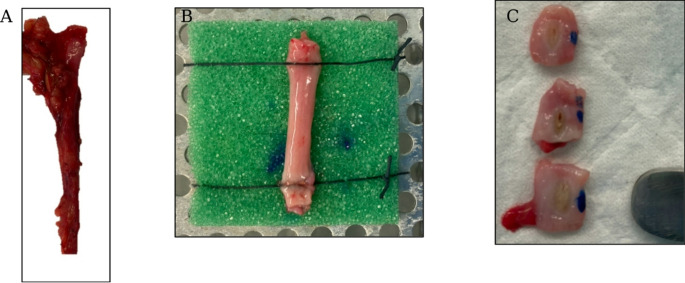
Caption: Preparation of human ureter Legend: Steps of specimen preparation, with fresh ureter transected at the renal pelvis immediately following nephrectomy (**A**). The specimen is then trimmed and divided longitudinally to expose the urothelium, and is secured on a custom sponge using silk sutures (**B**). Following treatment, the specimens are trimmed and the area of treatment marked with blue dye prior to placement in formalin (**C**)

Following approval from our institutional research and ethics committee (Ref: 22/20), human ureteric specimens were extracted from consenting adults at the time of radical nephrectomy for renal malignancy. Intra-operatively, the ureter was divided at the bifurcation of the common iliac vessels and extracted with the kidney. Immediately after nephrectomy, the ureter was trimmed of peri-ureteric fat and transected at the renal pelvis. (Fig. 1a) The specimen was stored in normal saline 0.9% (NS0.9%) at 37.5 °C for a maximum of one hour prior to testing. The ureter was incised along its longitudinal axis to expose the urothelium for testing. (Fig. 1b) Specimen thickness was measured at three points using a digital calliper, and the mean of the three measurements used for specimen thickness.

### Experimental set-up

**Fig. 2:**
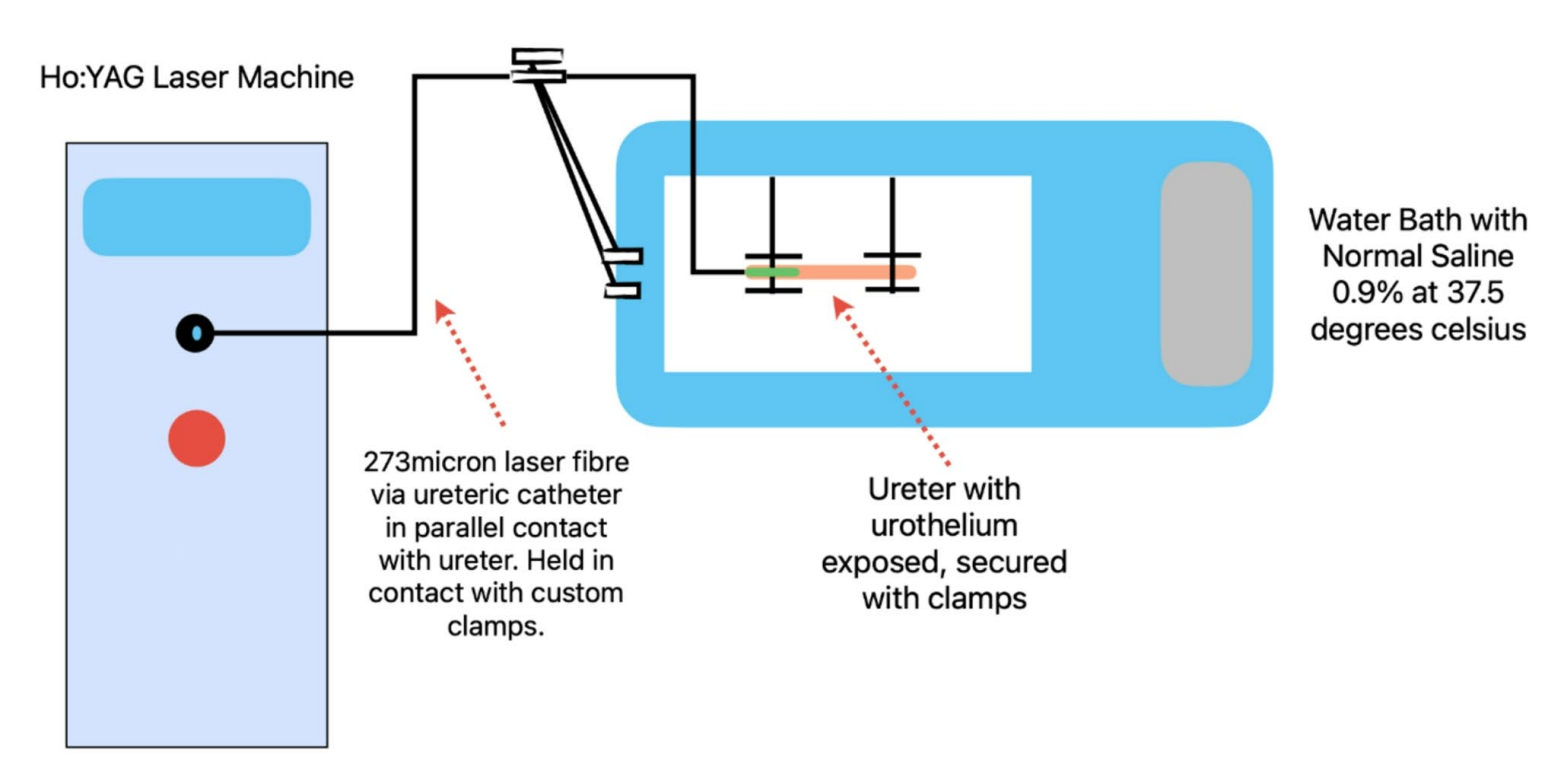
Caption: Simplified diagram of experimental rig set-up Legend: The specimen was secured as in Fig. 1B and placed in 37.5 C water bath with normal saline 0.9% A custom rig was used to hold the 273 μm Dornier Ho: YAG laser fibre which was passed through a 5Fr ureteric catheter and placed in direct contact parallel to the urothelium

A custom rig was developed for ex-vivo testing as in Fig. 2. Specimens were secured on sponge (Fig. 1b) and submerged in a water bath of NS0.9% at 37.5 °C. A 273 μm (micron) Ho: YAG laser fibre (Dornier MedTech Flexifiber©) was passed through a 6Fr (french) ureteric catheter and secured in place. The laser fibre was placed in direct contact with the urothelium in a parallel position to simulate the laser fibre position in the ureter when the patient is in the lithotomy position during ureteroscopy surgery.

### Laser testing

A Dornier MedTech Medilas H20 Ho: YAG laser was used for all experiments. Applied settings were 8 Hz (Hertz) and 800 mJ (millijoules), 10 Hz and 1000 mJ, and 5 Hz and 1500 mJ for durations of 5, 10 and 20 s. Following testing, specimens were analysed for macroscopic trauma. Blue dye (Cancer diagnostics Inc. Tissue Marking Dye©) was used to mark the area adjacent to testing and then immediately placed in formalin.

### Tissue processing

**Fig. 3:**
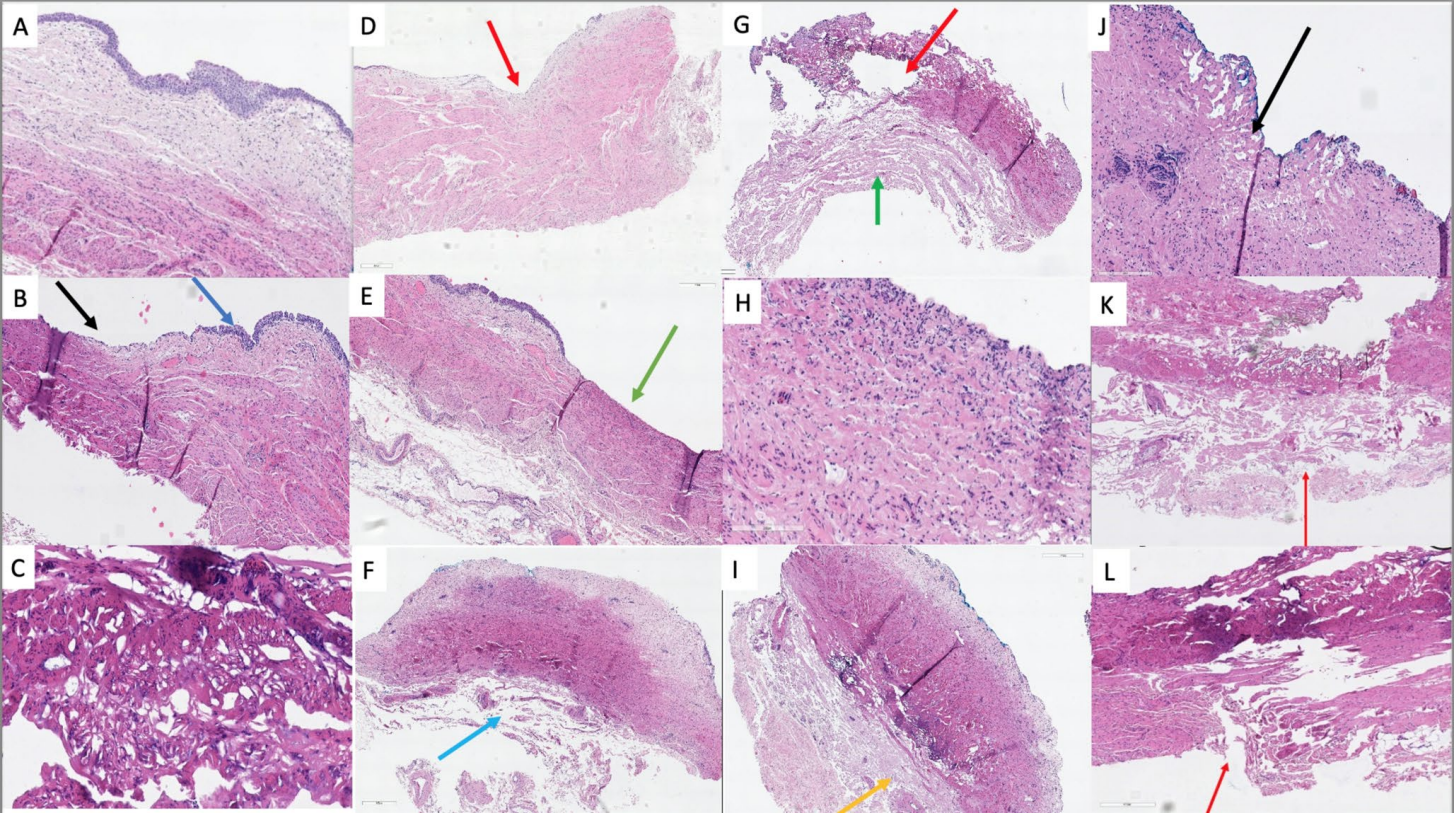
Caption: Representative images of human ureter following fixation, H&E staining and sectioning with image. **A**: Normal ureter anatomy at 400x of magnification. **B**: Illustrates the transition from normal (blue arrow) to abnormal interface (black arrow) following treatment with Ho: YAG laser. **C**: High power view showing changes (60x magnification) that are suggestive of more severe trauma to the ureter with blurring of cell nuclei with significant distortion and homogenisation of the tissue with prominent vacuolar change. **D-F**: Laser trauma in specimens treated at 8 Hz and 800 mJ at 400x magnification. **D** shows trauma extending to the urothelium only in a 0.9 mm thick specimen treated for 5 s. There is focal loss of transitional epithelium (red arrow), but normal epithelium either side of this. The underlying layers of the ureter appear normal, with no obvious cautery damage. **E** is representative of a 0.9 mm specimen treated for 10 s with laser trauma extending to the superficial muscularis with mild homogenisation and blurring of nuclei (green arrow). The remainder of the muscularis layers appear normal, with no significant cautery damage or vacuolar change. **F** demonstrates adventitial perforation (blue arrow) after 20 s of treatment in a 2 mm thick specimen. **G-I**: Treatment at 10 Hz and 1000 mJ is displayed in figures **G-I** at 400x magnification. Figure **G** is a representative image from a 2 mm specimen treated for 7 s with trauma extending from the urothelium to the muscle (red arrow) but preservation of the outer adventitia (green arrow). A representative high-power image(300x) from a 3 mm specimen treated for 10 s is seen in figure **H**, with abnormal nuclei in the lamina propria. However, the remaining three specimens treated for 10 s all had injury extending to the superficial half of the muscularis. Treatment of a 2 mm thick specimen for 20 s shows changes extending from urothelium through to full thickness of muscularis but with preservation of the adventitia (yellow arrow). Figures **J-L** show representative images following treatment at 5 Hz and 1500 mJ. Full thickness perforation or full thickness muscularis trauma was seen in all specimens tested at this setting excluding one. Figure **J** is a representative image at 200x magnification from a 1.7 mm specimen treated for 5 s showing full thickness muscularis changes but no perforation (black arrow). Adventitial perforation (red arrows) is seen in Figure **K** after 10 s of laser contact in a 2.1 mm specimen and Figure **L** after 20 s of laser contact in a 2.75 mm laser specimen (400x magnification)

Samples were fixed, sectioned, and stained using hematoxylin and eosin (H&E) prior to microscopic and histological evaluation (Fig. 3A-L)). All specimens were reviewed by a blinded histopathologist who graded the depth and extent of ureteral injury in each specimen. The level of iatrogenic trauma was defined by the deepest layer of tissue affected; urothelium, lamina propria, superficial muscularis, full thickness muscularis and adventitial perforation. Perforation was defined as trauma extending from the urothelium through all ureteric layers to include the adventitia.

### Data analysis

Tissue characteristics and treatment variables (contact duration, laser settings) were inputted into Microsoft Excel to allow generation of descriptive statistics. Chi-Squared Test and Students Paired T-Tests were used to evaluate for statistically significant relationships between tissue and treatment variables and full thickness ureter perforation. A *p* value of > 0.05 was deemed statistically significant.

## Results

### Specimen characteristics

In total, 31 human ureter samples were tested from 8 consenting adults at the time of radical nephrectomy for suspected renal malignancy. The characteristics of each specimen are summarised in Table 1. The mean specimen thickness was 2.2 mm (range 0.9–3.1 mm). A histological sample of tissue showing normal ureter unaffected by laser trauma and the transition from normal ureter to laser trauma is illustrated in Fig. 3A and B.

**Table 1:** Specimen characteristics, testing protocols and anatomical level of laser related trauma

Settings Used	*N*	Nephrectomy Side	Anatomical Location	Previous Urology Intervention	Patient Sex	Specimen Thickness (mm)	Laser Contact Duration (s)	Level of Injury	
8 Hz 800 mJ	1	Left	Proximal	-	M	0.9	5	Urothelium	
	2	Right	Proximal	Ureteroscopy	F	1.47	5	Muscularis	Inner half
	3	Right	Proximal	Ureteroscopy	F	1.47	5	Muscularis	Inner half
	4	Left	Proximal	-	M	0.9	10	Muscularis	Inner half
	5	Left	Proximal	-	M	0.9	10	Perforation	
	6	Left	Proximal	-	M	0.9	10	Urothelium	
	7	Left	Proximal	-	F	2	20	Muscularis	Full thickness
	8	Left	Proximal	-	F	2	20	Perforation	
	9	Left	Proximal	-	F	2	20	Muscularis	Inner Half
10 Hz 1000 mJ	10	Right	Proximal	-	M	2	7	Muscularis	Full thickness
	11	Right	Proximal	-	M	1	6	Perforation	
	12	Right	Proximal	-	F	3	5	Muscularis	Full thickness
	13	Right	Proximal	-	F	3	5	Urothelium	
	14	Right	Proximal	-	F	3	10	Muscularis	Inner half
	15	Right	Proximal	-	F	3	10	Lamina Propria	
	16	Right	Proximal	-	F	3.1	10	Muscularis	Inner half
	17	Right	Proximal	-	F	3.1	10	Muscularis	Inner half
	18	Right	Proximal	-	M	3	20	Muscularis	Full thickness
	19	Right	Proximal	-	M	3	20	Muscularis	Inner half
	20	Right	Proximal	-	M	2	20	Muscularis	Full thickness
5 Hz 1500 mJ	21	Right	Proximal	-	M	2.1	5	Perforation	
	22	Left	Proximal	-	F	1.7	5	Muscularis	Full Thickness
	23	Left	Proximal	-	F	1.7	5	Muscularis	Full Thickness
	24	Left	Proximal	-	M	2.75	10	Muscularis	Full thickness
	25	Right	Proximal	-	M	2.1	10	Muscularis	Full thickness
	26	Right	Proximal	-	M	2.1	10	Lamina Propria	
	27	Right	Proximal	-	M	2.1	10	Perforation	
	28	Left	Proximal	-	M	2.75	20	Muscularis	Full thickness
	29	Left	Proximal	-	M	2.75	20	Muscularis	Full thickness
	30	Left	Proximal	-	M	2.75	20	Perforation	
	31	Left	Proximal	-	M	2.75	20	Muscularis	Full thickness

### Laser settings and duration of contact

The depth of laser related injury ranged from urothelium only to full thickness perforation at all settings tested (Fig. 3; Table 1). The abnormalities noted on histological analysis include blurring of nuclei, homogenisation of tissue and vacuolar change (Fig. 3C). Overall, the abnormalities were focal and appeared milder at lower power and frequency settings (8 Hz/800 mJ) but more extensive at higher power (10 Hz/1000 mJ and 5 Hz/1500 mJ). This relationship was not statistically significant (*p* = *0.5*).

Testing settings of 8 Hz/800 mJ resulted in a range of injury from confined to the urothelium to full thickness perforation (Table 1; Figs. 3D-F). At short contact durations of 5 s, injury extended to the superficial level of muscle or urothelium only (Fig. 3D). At 20 s of contact, injury involved superficial muscle in one specimen, full thickness muscle trauma and full thickness ureter perforation (Fig. 3F). The specimens tested for 5 s all had thickness of 1.5 mm or less, compared to 2 mm at 20 s of treatment.

Laser settings of 10 Hz/1000 mJ were associated with injury, extending and ranging from urothelium only to full thickness perforation (Table 1; Fig. 3G-I). At the shortest duration of 5 s, injury varied from urothelium only to full thickness perforation (Fig. 3G). The specimen with full thickness perforation had a thickness of 1 mm. At 10 s injury extended to superficial muscle or lamina propria only, and at 20 s of contact duration injury involved muscularis in all cases (Figs. 3H-I).

The final settings of 5 Hz/1500 mJ had full thickness muscle injury or full thickness perforation in all specimens tested, excluding one which was confined to the lamina propria. (Table 1; Fig. 3J-L) Cautery changes extending to full thickness adventitia perforation and full thickness muscularis injury were seen at 5 s of contact at these settings (Fig. 3J). At 10 s of contact, injury extended to the lamina propria in one specimen, full thickness muscle trauma in one specimen and full thickness ureter perforation in the final specimen (Fig. 3K). At 20 s of contact, trauma extended from full thickness of the ureter in three specimens to perforation in one specimen (Fig. 3L). All specimens treated for 20 s at this setting were 2.75 mm thick (Table 1).

Overall, the level of injury seen with 5–10 s of treatment at any setting ranged from urothelium only to full thickness perforation depending on specimen thickness and laser power settings (Table 1). At 20 s of contact the minimal level of injury seen is superficial muscularis injury (*n* = 2, 20%), however the majority of specimens had full thickness muscle changes or perforation (*n* = 8, 80%).

### Specimen thickness

There was variation in the thickness of specimens with a range from 0.9 to 3.1 mm. No full thickness perforation was seen in specimens ≥ 3 mm (*n* = 8), even at 20 s of contact duration at 10 Hz/1000 mJ (Table 1). Specimens of ≤ 1 mm (*n* = 5) had levels of injury ranging from urothelium only to full thickness perforation. The remainder of specimens ranged from 1.1 to 2.9 mm (*n* = 18) with level of injury involving lamina propria, muscularis and full thickness perforation. There is a trend towards perforation with reduced specimen thickness, this was not statistically significant (*p* = *0.18*).

## Discussion

We present histological results of laser injury in human ureter using clinically relevant laser settings. Full thickness ureter perforation can occur at short contact durations and at low laser settings. The level of trauma produced by the laser is variable, with both superficial and extensive injury noted in different specimens at the same settings and duration of contact. Importantly, the thickness of ureteral specimens appears to be relevant in determining the extent of ureteral trauma following laser contact.

Our results show the impact of laser trauma to the ureter on a histological level with both thermal and mechanical injury. Thermal injury is noted as charring of tissue, blurring of nuclei, homogenisation of tissue and vacuolar change. Mechanical injury is noted as the destruction of tissue, with loss of tissue layers. The level of trauma varied from urothelium only to full thickness perforation through the adventitia. Similar injury types have been described in porcine studies with thermal laser injury noted as a discolouration rim of the entrance crater, the extent of which varied according to pulse rate [18]. Early studies in human ureter describe photothermal coagulation necrosis and fissures extending through ureteral layers [17]. Piergovanni et al. performed in-vivo porcine testing with delayed histological evaluation up to six days [16]. They described extensive lesions of partial or total necrosis of the porcine ureteral wall and urothelium with partial necrosis of muscle and lamina propria, and complete necrosis of urothelium [16]. This suggests that our results may underestimate the true extent of injury to the ureter with direct laser contact. In the real-life setting, the impact of inflammatory mediators and other clinical parameters may worsen the extent of injury seen.

The Ho: YAG laser has a good safety profile with a low risk of complications, however contemporary series have reported a stricture rate of up to 3% post ureteroscopy [19, 20]. This is higher than the rate of strictures seen with stones only, suggesting that the underlying pathology is related to treatment [20]. These strictures can be significant, with patients requiring further intervention including reconstructive surgery in 4.8% and nephrectomy in 1.7% [20]. Although the mechanisms for stricture formation are not fully understood and there are multiple potential contributing factors and ureteral perforation was identified as an independent significant risk factor for stricture formation [19]. It is theorised that the combination of periureteral fibrosis caused by urine extravasation and the inflammatory reaction caused by thermal injury leads to stricture formation [20]. Full thickness perforation was identified in *n* = 6 (19.4%) of our specimens, including at short contact durations and commonly used laser settings. We suggest that endourologists should exercise caution even at settings that are considered conventionally safe, as inadvertent contact between the laser and urothelium may have considerable clinical consequences for the patient.

As seen in Table 1, there was variation in the thickness of ureteric specimens tested ranging from 0.9 to 3.1 mm. Previous anatomical and imaging based studies have also reported wide ranges of ureteral thickness [21–23]. Ureter wall thickness can be measured on axial non-contrast computed tomography, the clinical relevance of this measurement is yet to be fully determined [23]. A recent meta-analysis showed a statistically significant correlation between wall thickness and spontaneous stone passage in the setting of urolithiasis, with higher rates of stone passage seen in thinner wall thickness measurements [22]. Ureter wall thickness has also been shown to impact the likelihood of successful external shockwave lithotripsy, with increasing thickness associated with lower success rates [22, 24]. In our results the extent of laser related injury varied according to specimen thickness, although not statistically significant there was a trend towards less severe injury in thicker specimens. No full thickness perforation was seen in specimens ≥ 3 mm, while in thinner specimens full thickness perforation was seen at 5 and 10 s contact. In the setting of stone impaction, the ureter may become inflamed, friable and prone to injury, so thicker tissue may also see significant trauma. Further studies comparing imaging based measurement of ureter wall thickness with pathological specimens and standardised imaging-based wall thickness measurement protocols are required. In the interim, surgeons should be aware that ureter thickness varies widely from patient-to-patient, and that this may carry with it a higher risk of ureteral perforation at commonly used laser settings with inadvertent laser contact.

Selection of the appropriate laser settings depends on many factors including stone type, size and location, surgeon preference and experience, and anatomical features. The ‘’optimal’ settings have yet to be defined and we chose to test commonly used laser settings that would represent the minimum and maximum expected power and frequency used in the ureter [9]. A previous review described “typical” fragmentation settings of 6–10 Hz and 1.0–1.2 J, with dusting settings of 0.2–0.4 J and 12–15 Hz [8]. A recent survey showed a preference for Ho: YAG for ureteral lithotripsy, with 80.8% of international experts surveyed choosing it over thulium fibre (TFL) or thulium-yttrium-aluminum-garnet (Tm: YAG) lasers [25]. The median maximum power used was 1000 mJ with a range of 500-1500 mJ, and a median maximum frequency of 15 Hz with a range of 6–50 Hz [25]. The settings we have chosen reflect commonly used settings in clinical practice and it is concerning to note that even at short contact durations (5 s) there can be injury extending to the muscularis, or indeed full thickness perforation.

Our testing was performed with a 273 μm fibre, the use of larger fibres may cause more significant injury at shorter durations or lower energy settings. Santa Cruz et al. evaluated Ho: YAG laser injuries in porcine ureter with a 365 μm fibre [15]. They describe ureter perforation at a minimum time frame regardless of frequency and power when in direct contact with the ureteric wall. At the minimum power setting of 1.0 W (200 mJ/5Hz) Ho: YAG produced a perforation within 1 s with a total energy of < 0.01 kJ when placed in direct contact with the ureteral wall at a ninety degree angle [15]. There are some limitations to these results; the laser was placed perpendicular to the outer aspect of the ureter (adventitia), which does not represent the typical clinical scenario. Other experiments with a 400 μm fibre on porcine ureter saw perforation with 0.02 kJ of energy applied, but the authors did not describe the duration of laser to urothelium contact [16]. Early testing in human ureter with a 400 μm fibre saw rapid perforation in < 1 s at 1500 mJ and 5 Hz, while in our specimens injury ranged from muscle to full thickness perforation at 5 s of contact at the same settings, and even at 20 s of contact there was only perforation in one of four specimens [17]. 

Ho: YAG has been the laser of choice for endourologists for over two decades with TFL representing the most common alternative due to its good dusting efficiency, low retropulsion and small fibre [25, 26]. In-vivo stone ablation in porcine ureters showed minimal urothelial damage with TFL or Ho: YAG on repeat inspection at 3 weeks and histological review of specimens [26]. Specific ex-vivo testing of TFL in direct perpendicular contact with porcine ureter showed a time to perforation of 7.9 s at 150 Hz and 1.8 s at 500 Hz [18]. Our results show full thickness perforation at 5 s of contact with 5 Hz and 10 Hz. Furthermore, we performed testing with the laser fibre parallel rather than perpendicular to the ureter in order to represent the position of the laser fibre in the ureter in conventional lithotomy position. This suggests the Ho: YAG laser may be safer than TFL, however comparative studies in human tissue are needed as well as further in-vivo testing to evaluate for other parameters that may contribute to tissue injury.

The limitations to our study include *ex-*vivo experiments and the contact durations sampled. Ex-vivo testing has its limits, as the impact of inflammatory mediators and tissue repair is absent. Intraoperative variations such as ureter oedema, inflammation, intra ureteric pressure, intra ureteric temperature and the manual pressure applied to the laser fibre could also affect the time to ureter perforation. We are limited with the in-vivo testing we can perform in human tissue, as it would not be appropriate to intentionally laser human ureter and remove it for pathological analysis. Nonetheless our ex-vivo findings contribute knowledge of Ho: YAG laser interaction with human tissue as current data relies mainly on animal studies [15, 16, 18]. Although providing useful information about the extent and type of injury associated with laser usage, the clinical application of these studies is limited as the laser was either applied to the ureter at a perpendicular angle or to the adventitia rather than the urothelium. The one previous study that evaluated laser injury in ex-vivo human tissue used a 400 μm fibre which is not commonly used in the ureter [17]. It is also unclear at what angle the laser was placed in contact with the ureter, and only a small number of specimens were tested (*n* = 7) [17]. Our experiments were performed with a 273 μm fibre which was in parallel contact to the ureter to simulate the position the laser fibre is in during ureteroscopy with the patient in the lithotomy position. A limitation to our study may include the contact durations chosen of 5, 10 and 20 s. We chose these as we considered them to show the extremes of laser contact that the ureter may be exposed to. Previous studies have focused on time to perforation using video or visual contact to confirm it, however it is not possible to determine microscopic perforation based on this basis alone and we wanted to evaluate the extent/depth of injury as opposed to purely full-thickness perforation [16, 17]. 

## Conclusion

This is the first study to evaluate laser injury in human ureter at commonly used laser settings with the Ho: YAG laser. Our results show that full thickness perforation and muscle injury occurs at commonly used laser settings. Further in-vivo testing is required to evaluate for other contributing parameters such as ureteric thickness and the impact of the inflammatory response in the ureter to laser contact and temperature. We suggest that the impact of laser contact may be more important than previously documented. In addition to stone parameters, measurement of ureteric thickness, with non-contrast CT, should be considered during pre-operative surgical evaluation of symptomatic urolithiasis patients. With the advent of new laser types, more powerful equipment and a move towards minimally invasive techniques we must advocate that the clinical implications of iatrogenic laser contact with human urothelium should not be forgotten.

## Data Availability

No datasets were generated or analysed during the current study.

## Abbreviations

Fr: French
H&E: Hematoxylin and eosin
Hz: Hertz
Ho:YAG: Holmium: yttrium–aluminium–garnet
IRP: Intra–renal pressures
IRT: Intra–renal temperature
mj: Millijoules
TFL: Thulium fibre laser
Tm:YAG: Thulium–yttrium–aluminum–garnet
nm: Nanometers
NS0.9%: Normal Saline 0.9%
µm: Micron
